# Norwegian nurses' perceptions of assisted dying requests from terminally ill patients—A qualitative interview study

**DOI:** 10.1111/nin.12517

**Published:** 2022-08-16

**Authors:** Hege Hol, Solfrid Vatne, Kjell Erik Strømskag, Aud Orøy, Anne Marie Mork Rokstad

**Affiliations:** ^1^ Faculty of Health Science and Social Care Molde University College Molde Norway; ^2^ Molde Hospital Molde Norway; ^3^ Norwegian National Centre for Ageing and Health Vestfold Hospital Trust Tønsberg Norway

**Keywords:** assisted dying requests, ethical challenge, interviews, moral distress, moral uncertainty, nurses' experiences, phenomenological hermeneutic design

## Abstract

This study explores the perceptions of Norwegian nurses who have received assisted dying requests from terminally ill patients. Assisted dying is illegal in Norway, while in some countries, it is an option. Nurses caring for terminally ill patients may experience ethical challenges by receiving requests for euthanasia and assisted suicide. We applied a qualitative research design with a phenomenological hermeneutic approach using open individual interviews. A total of 15 registered nurses employed in pulmonary and oncology wards of three university hospitals and home care in one municipality were recruited. Four themes emerged from the analysis: (1) unprepared for the request; (2) meeting direct, indirect, and nonverbal requests; (3) working in a gray zone, and (4) feeling alone and powerless. The study found that nurses were unsure how to handle such requests due to professional uncertainty about assisted dying. Working in an environment where the topic is taboo made nurses morally uncertain, and some perceived this as moral distress. The hospital chaplain played a significant role in providing support to these nurses.

## 1 INTRODUCTION

Nurses' perceptions of assisted dying requests may differ based on the legal status of assisted dying in their countries (Bruce & Beuthin, 2019; Elmore et al., 2018). In healthcare settings, assisted dying is a term that includes both euthanasia and assisted suicide according to a patient's voluntary and competent request, and the term corresponds to the Dutch definitions (Griffiths et al., 2008). Euthanasia is defined as an act (by a physician) to intentionally kill a person by injecting drugs, and assisted suicide is conducted (again, by a physician) by intentionally helping a person to end his or her life by providing drugs for self‐administration (Griffiths et al., 2008).

In jurisdictions where assisted dying is legal, empirical and ethical literature presenting nurses' experiences with assisted dying has been described (Pesut, Thorne, Greig, et al., 2019). However, in countries where assisted dying is illegal, knowledge about nurses' perceptions of this topic are limited (Wright et al., 2017).

The nursing profession has generally maintained a low profile in assisted dying discussions (Elmore et al., 2018; Terkamo‐Moisio et al., 2017). This may be related to the perception that the nursing profession has a limited role in assisted dying, and therefore, debates on the topic are considered outside the scope of the profession (Pesut, Greig, et al., 2020). Elmore et al. (2018) reported in a meta‐synthesis of qualitative research that nurses and physicians have traditionally positioned themselves differently in regard to the topic of assisted death. Physicians have been responsible for treatment decisions, while nurses have been described as patient advocates who facilitate open communication between the physician and the patient (Elmore et al., 2018). However, according to their 24‐h bedside attendance, nurses are more frequently involved in patients' suffering compared to physicians (Bruce & Beuthin, 2019; Terkamo‐Moisio et al., 2017).

Previous research has demonstrated that nurses are often the first professionals to receive requests for assisted dying (Pesut, Thorne, Stager, et al., 2019; Wilson et al., 2019). While some nurses find it quite common to receive such requests (Schiller et al., 2019), others are surprised and overwhelmed by patients' requests for assisted dying (Elmore et al., 2018). A recent review reported that nurses experienced mixed feelings about receiving assisted dying requests and reported difficulties finding the correct answers in line with their values and responsibilities as professional nurses (Cayetano‐Penman et al., 2021).

Regardless of the legal status of assisted dying, receiving such a request can be ethically challenging for any nurse (Norwegian Nurses Organisation, 2019). An ethical challenge refers to a value conflict entailing emotional and moral stress that is related to professional patient care (Storaker et al., 2017).

Using different approaches, previous studies have explored nurses' experiences in countries where assisted dying is illegal (Beuthin et al., 2018; Elmore et al., 2018). However, these experiences have been considered underrepresented in international research (Pesut, Thorne, Greig, et al., 2019). In Norway, assisted dying is controversial and illegal according to national law, and the political consensus, with some exceptions, is that there is no basis for making changes in this legislation (Kleiven et al., 2020). Nurses have professional, ethical, and personal responsibilities for their own actions and assessments, and they should be familiar with the legislation that regulates the nursing profession in accordance with the Health Personnel Act (1999). The Professional Ethical Guidelines (Norwegian Nurses Organisation, 2019) make it clear that Norwegian nurses should not contribute to euthanasia and assisted suicide. In a review of the available literature undertaken in planning this study, it appeared that Norwegian nurses are invisible in the research literature and in public discussions about assisted dying. Information regarding their perceptions of requests for assisted dying is limited (Gustad et al., 2020; Hol et al., 2022). However, a recent survey among Norwegian nurses revealed that 58% of 205 responding nurses had received requests for assisted dying from terminally ill patients (Hol et al., 2022). This finding indicates a need for knowledge about how these nurses perceive such requests for illegal assistance. Hence, this study aimed to explore Norwegian nurses' perceptions of assisted dying requests from terminally ill patients by answering the following research questions: (1) How do nurses experience receiving requests for assisted dying? and (2) What are their perceptions of moral uncertainty and distress when receiving requests for such an illegal action?

## 2 MATERIALS AND METHODS

### 2.1 Study design

This study employed an exploratory qualitative design. Individual open interviews were conducted to grasp the meaning of nurses' perceptions of receiving a request for assisted dying (Patton, 2014; Polit & Beck, 2020). Inductive data were analyzed using a phenomenological hermeneutic method inspired by (Lindseth & Norberg, 2004). While nurses may experience ethical challenges related to patient care, they are not always able to explain and justify their ethical thinking (Lindseth & Norberg, 2004). To gain an in‐depth understanding of their perceptions, we asked participants to relate incidences from their clinical work.

### 2.2 Sample and settings

The participants were recruited based on their feedback to the question, “Have you received an assisted dying request from a terminally ill patient on your ward?” on an electronic anonymous survey created by the research group. Those who answered “yes” received an electronic invitation to participate in this study. Six participants from pulmonary and oncology wards responded to the invitation by sending the first author an email. To ensure variation of participants' perceptions, additional participants were recruited from a home‐care department and from oncology and pulmonary hospital wards with assistance from the heads of those wards. The hospital wards were selected because they provided care and treatment for terminally ill patients who were suffering from chronic and medical conditions. We assumed nurses in the home‐care district provided the same treatment. When estimating the sample size, the saturation of data was considered achieved in the 13th interview. However, we decided to conduct the last two interviews with participants who had already accepted the invitation to be included (Malterud et al., 2016). Hence, the final sample comprised 15 nurses, 3 men and 12 women, from one municipal home‐care department and three university hospitals across Norway. Criteria for inclusion included being a registered nurse, working full time at the bedside, proficiency in Norwegian, and having received an assisted dying request in the past and/or present. The sample characteristics are presented in Table 1.

**Table 1:** Characteristics of the participants

Total	*N* = 15
Gender	
Female	12
Male	3
Age (years)	
25–29	4
30–34	3
35–39	1
40–44	3
45–49	1
50–54	0
55–60	2
60+	1
Education	
Bachelor's degree	10
Bachelor's and formal continuing education in palliative care	5
Department	
Oncology ward	4
Pulmonary ward	6
Home‐care district	5
Number of years working	2–30

### 2.3 Data collection

We completed the inductive data collection from January to July 2018. An open interview approach (Patton, 2014) was used. A total of 13 interviews were conducted face‐to‐face at the participant's workplace or in public libraries, and two participants were interviewed by telephone. In the interviews, participants were encouraged to speak freely. Moreover, they were given time to develop their story with minimal interruption. A deeper reflection was encouraged to elaborate the story to elicit in‐depth details of participants' perceptions. All the interviews were audio recorded.

To ensure a phenomenologically inspired entrance to exploring the study's aim, the first question asked was, “Please tell me about a situation in which you received a request for assisted dying from a terminally ill patient.” Clarifications and elaborations were secured through the mirroring of statements, and follow‐up questions were asked (Patton, 2014).

The first author conducted and transcribed the interviews verbatim (Polit & Beck, 2020). The transcripts included laughter, tears, and pauses, and this information was marked to capture the implications as well as the interviewees' feelings. The transcriptions were validated against the audio files. All transcribed data were deidentified, and each interviewee received a number to ensure participants' confidentiality. The interviews lasted between 60 and 140 min, and the transcribed data material totaled 616 written pages. Transcripts were not returned to the participants for comments.

### 2.4 Data analysis

A phenomenological hermeneutic analysis proceeded through a dialectical movement between the understanding and interpretation of the text. It consisted of three methodological phases: naive understanding, structural analysis, and comprehensive understanding (Lindseth & Norberg, 2004).

The first phase of the analysis included a naive understanding, and each transcribed interview was read several times by the first author in its entirety chronologically. The content was validated and interpreted with an open mind to gain a sense of the entire data material. Furthermore, suggestions for themes to include in the structural analysis were discussed by the research team.

In the second phase of the analysis, several thematic structural analyses were conducted. A theme is an abstract meaning unit that brings meaning and identity to a present experience (Lindseth & Norberg, 2004). A meaning unit consisted of one sentence related to a larger text unit. To capture the meaning of the experiences, the meaning units were formulated as condensed descriptions that expressed the content of the participants' perceptions without changing the meaning of the content. All meaning units from each interview were identified, sorted, and reflected upon. Then, a transversal analysis was conducted, and meaning units were abstracted to themes as illustrated in Table 2. During this phase, the phenomenological perspective shifted from an individual to an objective interpretation of the participants' perceptions. Based on the aim of the study and the naive reading, all condensed meaning units were read and reflected on by the research team. Abstraction of the themes involved reflection on the meaning content in terms of similarities and differences.

**Table 2:** An example of structural analysis (Lindseth & Nordberg, 2004)

Naïve reading		
The nurses were surprised to receive a request for assisted dying and found this difficult to interpret and to find the right words in their response. The requests occurred when the nurses were alone with the patient, and they felt powerless because they did not know how to handle the situation properly. The nurses experienced relieving patients' suffering as difficult.		

**Meaning unit**	**Condensation**	**Theme**
I never thought I would receive a request like that. I thought, in Norway, you do not get questions like that. Because we do not perform assisted dying in Norway. I never considered what to answer, and I have never considered my thoughts on it.	I was surprised and did not know how to handle an illegal assisted dying request.	Unprepared for the request
…the patient indirectly asked, “I cannot live like this anymore. Animals are treated better than I am. If I were a dog, I would receive an injection or a bullet in my forehead ….” I mean, this statement is indirect.	The patients expressed the request in an indirect way by comparing themselves to how animals are treated better and euthanized when their suffering cannot be relieved.	Meeting direct, indirect, and nonverbal requests
I was a recent graduate, and I was not sure how much of morphine I could inject. The patient's family stood around the bed. I was thinking, I know how much morphine I have injected. I can give a little more, but then it will be some time before I can give more. I was very stressed and thought, “Have I done it right?”	I was stressed not knowing how much of the morphine the patient could endure.	Working in a gray zone
I felt powerless every single time because I wished patients could die faster. I could have helped, but I am not allowed. Then, you just must watch! I think it is unworthy. It goes against my moral principles. I have learned that the patient should die with dignity, and dignity involves being pain relieved.	I felt powerlessness as I was not able to relieve the patient's suffering.	Feeling alone and powerless

In the last phase, comprehensive understanding of the discussion, we summarized the themes and reflected on their relation to the study's aim. Here, we critically validated the naive understanding and structural analyses in their entirety while considering ethical theories, legislation, and research.

The analyses were performed by five researchers, four women and one man, all of whom had clinical experience.

### 2.5 Ethical considerations

The study was approved by the Norwegian Regional Committee for Medical Research Ethics Central Norway reference number 2017/1131/REK midt, and the Norwegian Center for Research Data (NSD) reference number 997338 in 2017. The study was conducted in accordance with the Declaration of Helsinki. Additionally, the ethics committees of the university hospitals and home‐care departments granted approval for the study. Before the interview, a declaration of consent was emailed to each participant. The declaration was repeated in the interview setting. To avoid misunderstanding, a definition and examples of assisted suicide, euthanasia, and treatment limitations were included in the information letter and during the interviews. All participants provided written informed consent. Since the topic could be perceived as controversial, the interviewer paid particular attention to participants' emotional reactions during the interviews. To protect the anonymity of the small number of male participants, we refer to all participants as female when presenting the findings.

### 2.6 FINDINGS

The participants described two to five stories each about receiving a request for assisted dying. Their narratives varied according to the degree of the detailed description. Taken together, they provide an open and rich collection of data.

The structural analysis revealed the following four themes presented as the study findings: (1) unprepared for the request; (2) meeting direct, indirect, and nonverbal requests; (3) working in a gray zone; and (4) feeling alone and powerless.

### 2.7 Unprepared for the request

Almost all participants stated that they were not mentally prepared for their first assisted dying request. Even incidents with requests that occurred several years ago had not been forgotten as they had made a significant impression on participants. They explained that they received the request when they were completely alone with the patient and had no support from colleagues. They described their reactions as surprised, shocked, and speechless. One of them explained, “Inside my head, there was complete chaos…I was completely set out” (P2).

A few participants talked about their desire to escape from the situation by leaving the patient's room immediately because they experienced the situation as intense and uncomfortable. The participants related their lack of preparedness for such requests to not being familiar with the fact that patients could request assisted death on the ward: “I never thought I would get such request in Norway because we do not perform assisted dying” (P2). During the interviews, several participants reflected on why they had received this request without finding a satisfactory explanation. One participant said, “I actually have no idea why he asked me … No idea!” (P6).

A common perception expressed by almost all the participants was their uncertainty related to how to respond to the request. One participant explained: “The whole situation was uncomfortable because you are asked about something that is illegal. You read about it in the media saying, ‘It is not allowed!' (…) It is scary, and you do not want to talk about it with the patient” (P2). Participants found it difficult to find the right words when responding to the patient and did not know how to answer. A few participants were more comfortable about how to respond to such a request: “When I received the request, I said to him, ‘No, I am sorry, I cannot do that. (…) I understand your decision and why you ask, but I am afraid I cannot help you to end your life” (P15).

### 2.8 Meeting direct, indirect, and nonverbal requests

During the interview, the participants used the terms “direct,” “indirect,” and “nonverbal” to refer to the way the request was communicated by patients. A direct request was formulated in a way that left no room for misunderstanding, for example, “Please, can you use the medicine you administer to take my life?” (P11). Several participants described that, often, terminally ill patients would ask for an overdose of a morphine injection because they knew that nurses had the knowledge to administer this medication. Participants also explained that patients have a perception that nurses have uncontrolled access to medication.

Moreover, all the participants referred to indirect requests, which occurred more frequently and were perceived as the most difficult to interpret. For example, a participant referred to a patient who said, “I cannot live like this anymore. Animals are treated better than I am. If I were a dog, I would receive an injection or a bullet in my forehead” (P1). She explained that the patient saw no other way to end his suffering and could not imagine living like this for the rest of his life. Another participant shared a similar example, that she interpreted as a direct request.

Several participants reported that patients made requests in a nonverbal manner. These requests were not easy to understand, and the participant's interpretation depended on the relationship to the patient. Receiving requests from patients who could not communicate verbally was complicated, as illustrated by the following quote:

He hid the PEG probe and then tried to rip it out. He did not want medication, food, intravenous, or anything orally but turned his head away. I think this was his way of saying, “I do not want this life!” (P6).

A few participants perceived that a patient was asking for help by stockpiling medication. A participant described how one of her patients had suggested that they could hide medication in the bedside drawer to be able to help the patient end her life:

She started crying as I came in the door: “I simply do not know how much longer I can handle this. I am so tired!” She said she had a plan for how we could hide doses of morphine in the drawer until we had enough for an injection to let her fall asleep quietly and calmly (P15).

When receiving a request for assisted dying, some participants observed and referred to a darkness in patients' eyes. One of the participants explained that the look in the patient's eyes had to be interpreted along with how the patient uttered the request and the pitch in his or her voice. This participant explained that she interpreted the patient's signal as a trustworthy request. However, several participants found it difficult to interpret what patients communicated with their eyes. For example, one said, “It is inexplicable what those eyes tried to tell me” (P1).

### 2.9 Working in a gray zone

The participants expressed uncertainty about their ability to interpret requests for assisted dying and with working in accordance with the penal code. Their perception of experiencing a dilemma between being unable to relieve a patient's suffering, on one hand, and their obligation to follow their statutory duty, on the other: *“*It's like standing there with the Norwegian Penal Code above my head, pecking at my shoulder” (P2). Almost all the participants asserted that, in some situations, they had been afraid to administer legally prescribed morphine as it could hasten a patient's death. For example, one participant stated, “You always become unsure if you give too much morphine because you are afraid it will end in assisted dying” (P12).

Participants mentioned that working in such a gray zone could sometimes lead to underdosing of pain medication due to lack of knowledge and fear of doing something wrong. Some participants were afraid that they might inadvertently end a patient's life as a result of the “last injection.”

### 2.10 Feeling alone and powerless

All of the participants were convinced that they were the only ones on the ward who had received a request for assisted dying from a patient. Being asked to do something illegal made them feel vulnerable. Moreover, they lacked support from their colleagues in these situations as such requests were not shared. One participant explained:

This request is not talked about on the ward because one is afraid of being stigmatized. (…) You do not know what the other person is thinking. (…) The reason why we, the nurses, do not talk about this is that we do not have experience or knowledge. (…) Assisted dying is a difficult subject and a taboo subject. Nobody wants to step forward and say what they think about this subject (P9).

The participants found the requests to be private and associated with patient confidentiality. Hence, assisted dying requests were perceived as taboo since the participants did not want to violate the patients' confidentiality. All the participants explained that, even though nurses and physicians worked with the shared goal of alleviating the patient's suffering, they never talked about their perceptions of receiving an assisted dying request. One explained, “Assisted dying is something I never discuss with a physician” (P13).

Several participants mentioned that they never documented the patient's request in the medical records because of the possible consequences:

I thought, since I am not able to give an answer [to the patient], I do not want the answer to be read either. I could receive critical questions from my colleagues (P2).

However, a few participants said that they had discussed a patient's request with the hospital chaplain. These participants explained they trusted the chaplain and spoke without fear of stigmatization and rumors on the ward among colleagues. Several participants revealed that their relationship with the patient became more difficult after the request.

I felt powerless every single time because I wished patients could die faster. I could have helped, but I am not allowed. Then, you must just watch! I think it is unworthy. It goes against my moral principles. I have learned that the patient should die with dignity, and dignity involves having their pain relieved (P9).

The feeling of powerlessness was associated with the difficulty of relieving the pain and suffering of terminally ill patients. In situations where the participants were unable to relieve the patient's suffering, they felt that they were the only ones who comforted and cared for the patient in his or her suffering. Three participants explained that experiencing powerlessness over a long period of time had led them to decide to quit their jobs.

## 3 DISCUSSION

This study explored Norwegian nurses' perceptions of receiving requests for assisted dying from terminally ill patients. The participants described such requests as ethically challenging as they were unprepared and felt alone in those situations where they had to deal with them.

There is limited qualitative research on nurses' perceptions of receiving requests for assisted dying in countries where it is illegal. However, even studies conducted in locations where assisted dying is legal have found that nurses may perceive moral and ethical dilemmas upon receiving such requests (Bruce & Beuthin, 2019; Cayetano‐Penman et al., 2021; Wright et al., 2017).

### 3.1 Privatization of a professional dilemma

Participating nurses in this study interpreted the dying request as private because they believed that it was confidential and solely tailored to themselves. Based on this assumption, the request remained confidential between the nurse and the patient and was not discussed in the interdisciplinary team. Talking openly about assisted dying was considered taboo on the ward, a finding that is in line with a recent study by Mills et al. (2020) that discussed assisted dying as a taboo theme. They argued that as nurses believed that they would violate their duty of confidentiality if they conveyed the request, they decided not to discuss this request with colleagues. This decision could indicate a misinterpretation of the legislation of confidentiality (Mills et al., 2020). The nurses in our study felt a strong sense of confidence from the patients that they did not want to damage. According to the Health Personnel Act in Norway, healthcare professionals should share confidential information in professional communities, where the purpose of sharing it is to support healthcare personnel in making appropriate decisions related to patient healthcare (The Health Personnel Act, 1999). We consider dying requests to be important information that needs to be shared and discussed with colleagues to be able to meet the patients' needs.

### 3.2 Professional powerlessness

The participants in our study felt unprepared for assisted dying requests and were surprised and overwhelmed when they received them. They found the request to be difficult because they had no expertise in interpreting and managing the situation. De Bal et al. (2006) reported that some nurses perceived requests as a directly or indirectly formulated request, and they were not always able to understand patients' requests, especially when a patient used nonverbal and behavioral cues to convey a request (Hudson et al., 2006; Pesut, Thorne, Greig, et al., 2019; Wright et al., 2017). The findings of our study indicate that the Norwegian nurses had the same experiences. They expressed discomfort and ethical uncertainty, and they felt unable to help the patient. Similarly, Beuthin et al.'s (2018) study revealed that nurses who rarely receive requests were surprised to learn that they are perceived to be morally and ethically challenging.

The participants in our study did not know who to talk to or who to trust to discuss the request they had received. In this situation, a few nurses consulted their hospital's chaplain as a trusted, conventional colleague with whom to discuss their moral and ethical dilemmas. This finding indicates that hospital chaplains may play a major role in giving nurses significant support when they are feeling uncertain in these situations. We were not able to identify similar findings from previous studies.

The present study revealed information about nurses who lacked knowledge regarding the distinction between relieving pain and shortening life according to Norwegian legislation and guidelines (The Health Personnel Act, 1999; The Penal Code, 2005). The nurses feared that they might be the one to give the “last injection” that would shorten a patient's life and potentially be misinterpreted as euthanasia. The participants used the term “last injection,” which is a concept used synonymously with the term “double effect.” McIntyre (2012) explained that a double effect is when medication is used to relieve a terminally ill patient's pain and suffering and may, as a side effect, hasten death. The intention is to achieve relief of symptoms and not euthanasia. The nurses in our study felt trapped in a situation that could not be resolved, making them feel vulnerable and powerless as they were unable to relieve suffering. This finding is comparable with that of the study by Gustad et al. (2020) suggesting that nurses' lack of medical knowledge might lead to underdosing and, thereby, the unnecessary suffering of terminally ill patients.

In our study, all the nurses were unsure about how to manage a request for assisted dying, as well as the topic itself, due to a lack of knowledge and training in their bachelor's degree nursing education. Since they were trained to care for the dying patient, they found it incomprehensible that they had not received knowledge about patients' requests to hasten their death. This lack of knowledge about assisted dying requests from terminally ill patients might have consequences for nursing practice. In contrast, in a study conducted in Canada, research has revealed that nurses with relevant knowledge to help patients who requested assisted dying were able to talk openly with the patient about the request (Bruce & Beuthin, 2019; Elmore et al., 2018; Wright et al., 2017). An earlier study by Hudson et al. (2006) asserted that nurses have an obligation to respond professionally and compassionately to the patient's request for assisted dying, to assess each request, and to identify treatable problems or concerns.

### 3.3 Moral uncertainty and moral distress

Ethical and moral decisions in response to receiving a request for assisted dying may produce conflicts for nurses that can lead to moral uncertainty and moral distress. A concept analysis of moral uncertainty and moral distress performed by Dorman and Bouchal (2020) explained that moral uncertainty includes a lack of probable and preferable outcomes. Nurses experienced internal conflicts such as feelings of guilt, self‐questioning, and frustration. Moral distress includes the inability to take the right action, feelings of powerlessness due to external forces that prevent nurses from acting, and the perception of unnecessary suffering that is not in the best interests of the patient. There is significant overlap between these two concepts, thus influencing how nurses may respond to situations where patients request assisted dying (Dorman & Bouchal, 2020).

In our study, the participants perceived feelings of frustration, powerlessness, and vulnerability when they received the request, knowing they could not fulfill the patient's wishes. They found this situation morally difficult because they were alone with a suffering patient who asked them to help them die, a patient who was “not allowed” to die because euthanasia is illegal. The participating nurses experienced this request as a personal dilemma for which they were not existentially prepared. There were no routines or guidelines for how to handle requests for assisted dying on the ward. Furthermore, they were not aware of how to observe and recognize requests for euthanasia and assisted suicide. The participants who had received such a request kept it secret, as the lack of communication about the topic on the ward made them perceive the request, as well as any discussion about it, as taboo. All this made them feel vulnerable and uncertain because they did not know how to resolve the situation. Working alone in moral uncertainty became a personal dilemma for which they had not been prepared. Similarly, Pesut, Thorne, et al. (2020) described Canadian nurses' emotional and moral conflict as an ongoing journey of moral uncertainty in regard to participating in assisted dying or not. Their study revealed that even when assisted dying is legal, nurses may perceive this act as ethically challenging.

According to Dorman and Bouchal (2020), moral uncertainty implies an inability to decide which course of action to take or to know what outcome is preferable. Our study shows that the majority of the participants lost control and became morally uncertain when receiving the request. They were unable to know and decide on what action to take, and additionally, they experienced uncertainty about how to relieve the terminally ill patient's suffering. Dorman and Bouchal (2020) asserted that moral uncertainty around end‐of‐life outcomes often results in nurses' internal conflicts. They may become less verbal in situations of uncertainty and struggle internally with questions about the correct course of action (Dorman & Bouchal, 2020).

The majority of the participants in our study perceived moral uncertainty because they were not prepared for a request for assisted dying. The participants' moral uncertainty could be caused by the absence of a professional arena in which to discuss ethical challenges. However, we found that the hospital chaplain was an important person for providing support and the only person they trusted on the ward because the chaplain's duty of confidentiality allowed them to feel free to discuss moral issues without feeling stigmatized.

According to Dorman and Bouchal (2020), moral uncertainty may lead to moral distress when it becomes too difficult and extends over time. Several participants perceived moral distress based on situations that ended in a poor patient outcome. Three participants in our study had decided to leave their profession because of the sense of powerlessness at being unable to act and provide patient care in a professional manner. Dorman and Bouchal (2020) asserted that moral distress in the context of palliative care might be unavoidable as nurses often experience conflicts of conscience in caring for patients. They argue that uncertainty may cause nurses to question themselves as professionals. A general acceptance of uncertainty as a part of professional practice can lead to indifference in regard to both resolving moral dilemmas and acquiring new knowledge (Dorman & Bouchal, 2020).

The nurses in this study came from university hospitals and home‐care districts all over Norway, which is a strength. However, we see in retrospect that we could have made other priorities in the inclusion criteria such as small hospitals and nursing homes. The sample in this study is homogeneous, and hence, the findings must be interpreted with caution.

Rigor and trustworthiness were achieved through the description of the context, the number of participants, and the findings according to this study's aim (Polit & Beck, 2020). The first author conducted and transcribed the interviews according to Polit and Beck's (2020) recommendations. The group of researchers, all with experience in clinical practice, collaborated in discussing and interpreting the themes drawn from the analysis. The researchers provided transferability through detailed descriptions of findings and verbatim quotes.

Despite limitations, the study, through its participants, has contributed to the Norwegian empirical literature by providing insights into nurses' perceptions of receiving assisted dying requests. Their stories from clinical practice are subjective and told the way they perceived them. This topic is important for the development of clinical knowledge about assisted dying in nursing practice since research on this topic has been absent in Norway.

## 4 CONCLUSION

The study reveals that Norwegian nurses need to be able to talk freely about moral and ethical challenges they perceive in clinical practice when receiving assisted dying requests. It is important to focus on this topic, and nurses need to facilitate an open discussion among their nursing colleagues, leaders, physicians, and hospital chaplains. Sharing moral uncertainty and moral distress may strengthen and promote nurses' competence and reduce the taboo of the concept of assisted dying in clinical practice.

Furthermore, nurses should initiate an open and professional focus in the public discussion of assisted dying, a discussion that should illuminate the topic from multiple perspectives without the main intention of legalizing assisted dying.

The findings emphasize the need to meet nurses' moral uncertainty and distress when they receive requests for an illegal action such as assisted dying. Collaboration with other professionals including physicians and leaders of various religious denominations should be further explored. The fact that nurses receive assisted dying requests makes it important to prepare them for how to address such requests and, in the process, increase their competence. We recommend developing guidelines for nurses about how to handle assisted dying requests; such guidelines should include other professionals who should be notified of such requests so that the nurse is not alone in addressing such a request.

Future research should focus on nurses' professional competence regarding how to communicate with patients who make an assisted dying request. Such research should also aim to identify ways to strengthen a team approach and communication to relieve pain and suffering and highlight and advance knowledge surrounding ethical and legal concerns at the end of life.

## CONFLICT OF INTEREST

The authors declare no conflict of interest.

## Data Availability

Research data are not shared.
